# Socioeconomic differences in adolescents’ smoking: a comparison between Finland and Beijing, China

**DOI:** 10.1186/s12889-016-3476-0

**Published:** 2016-08-17

**Authors:** Yang Liu, Mei Wang, Jorma Tynjälä, Jari Villberg, Yan Lv, Lasse Kannas

**Affiliations:** 1 School of Physical Education and Sport Training, Shanghai University of Sport, 200438 Shanghai, China; 2 Shanghai Research Centre for Physical Fitness and Health of Children and Adolescents, Shanghai University of Sport, 200438 Shanghai, China; 3 Mass Sport Research Centre, China Institute of Sport Science, 100061 Beijing, China; 4 Research Centre for Health Promotion, Department of Health Sciences, University of Jyvaskyla, 40014 Jyväskylä, Finland; 5 Zhejiang Institute of Sport Science, 310004 Hangzhou, China

**Keywords:** Adolescent, Smoking, Family Affluence Scale, Socioeconomic inequalities

## Abstract

**Background:**

Various studies have demonstrated the associations between socioeconomic status (SES) and health and health behaviour among adolescents. However, few studies have compared the socioeconomic difference in adolescent smoking between countries with different stage of smoking. The purpose of this study was to examine and compare the relationship between socioeconomic status (SES) and adolescent smoking in Beijing, China and Finland through the Health Behaviour in School-aged Children (HBSC) study.

**Methods:**

The data used in this study were derived from the Chinese HBSC linked project survey 2008 in Beijing and the Finnish HBSC survey 2006. The final sample included 2005 Chinese and 1685 Finnish 15-year-old schoolchildren. The associations between Family Affluence Scale (FAS), as the SES measure, and adolescents’ smoking behaviour, including ever smoked, weekly smoking and the early onset of smoking were examined separately in two countries through binary logistic regression.

**Results:**

Compared to students from the high FAS group, Chinese boys from the low FAS group were more likely to report having ever smoked (*OR* = 2.12, 95 % *CI* = 1.49–3.01) and being early onset of smoking (*OR* = 2.17, 95 % *CI* = 1.44–3.26). Finnish girls from the low FAS group were more likely to report being weekly smokers (*OR* = 1.68, 95 % *CI* = 1.07–2.65). No significant difference was found for Chinese girls and Finnish boys.

**Conclusions:**

This study indicated different patterns of socioeconomic difference in smoking between Chinese and Finnish adolescents by gender and by smoking behaviour, which suggests that socioeconomic inequalities in smoking are different among adolescents in countries with different stage of smoking. Country specific policies and interventions for different target groups should be encouraged and designed for reducing the prevalence of adolescents’ smoking.

## Background

Globally, it is estimated that 150 million young people currently use tobacco [1]. The prevalence of smoking in adolescence varies widely around the world. Nearly three decades ago, Lopez et al. [2] proposed a four-stage model of cigarette smoking in a country (stage 1-low smoking prevalence in men and very low in women; stage 2-smoking prevalence in men rapidly increases while it increases slowly in women; stage 3-smoking prevalence in men decline and in women peaks and then begins to decline; stage 4-smoking prevalence in both men and women continue to decline. In most high-income countries, the rate of smoking is keeping steady or decreasing). It has been reported that although there are decreases and increase in smoking of Finnish adolescents from the 1970s to the 2000s, overall smoking behaviour has declined since 2000 [3]. For instance, according to the Adolescent Health and Lifestyle Survey (AHLS), experiencing smoking and daily smoking have decreased among Finnish adolescents during the study period 1977–2011 in all age groups and both genders [4] and the decreasing trend continued also over the past 2 years [5]. These facts demonstrated that at present, Finland has reached the final stage at which the overall prevalence of smoking is decreasing.

Contrary to high-income countries, with westernization and economic development, adolescents’ smoking is increasing and becoming more popular in developing countries [6]. The prevalence of adolescents’ smoking has increased dramatically in China since the 1990s [7]. In a Youth Risk Behaviour Survey in Guangzhou, the biggest city in southern China, nearly one fifth of students aged from 12 to 19 years reported they had tried smoking [8]. According to a recent meta-analysis of smoking behaviour among Chinese adolescents, the estimated prevalence rate of lifetime smoking (ever smoked) varied within a narrow range (39–46 %) for males and progressively increased from 2 % in 1981–1985 to 19 % in 2001–2005 for females [9], which indicated that China has reached the third stage of smoking. Though the absolute percentage of young smokers in China is still less than in many developed countries, and varies across age, gender and area, the increasing trend of cigarette smoking in China should not be neglected since young smokers are more likely to become habitual smokers in adulthood [10].

Smoking has been influenced by many factors. Among them, socioeconomic factors play an important role in smoking. In Western countries, numerous studies regarding socioeconomic inequalities and smoking have been done in adult populations and the results indicate that people from lower socioeconomic status (SES) groups, defined by income, education, occupation or other SES measures, are more likely to smoke [10–13]. In contrast to studies on adult populations, the literature addressing the socioeconomic inequalities in smoking among adolescents is limited and the literature revealed inconsistent results. Several studies have showed that there is an association between smoking and SES during adolescence in some European countries [14, 15] and in developing countries like Ghana [16], while other research have indicated that there was no relationship between SES and adolescents’ smoking [17, 18]. Moreover, a study analysing trends in socioeconomic differences and smoking among German schoolchildren revealed that family affluence had only a weak effect on regular smoking [19]. The latest international report from the Health Behaviour in School-aged Children (HBSC) study revealed that the patterns of socioeconomic inequalities in smoking over 30 countries among 15-year-olds are different [20]. In addition, Pförtner and his colleagues recently found that the difference in smoking prevalence between rich and poor is greater in more affluent countries after investigated the association between family affluence and adolescent smoking in 33 European countries, Israel and Canada [21].

These inconsistent results were also observed in studies concerning Chinese and Finnish adolescents. Based on the HBSC study, Schnoher and her colleagues [15] found that Finnish adolescents from the low affluent families were more likely to be daily smokers. The results from the European School Survey Project on Alcohol and Other Drugs (ESPAD) survey, however, showed a minor but persistent difference in smoking among adolescents by parent education that young smokers whose parents have the low education level were more than those young smokers whose parents have the high education level [22]. Furthermore, Paavola et al. [23] demonstrated that there was no relationship between SES and adolescents’ smoking. Among limited studies concerning Chinese adolescents reported, positive associations between daily smoking and SES were not found in Guangzhou Youth Risk Behaviour Survey [8], but were found in the China Adolescent Behavioural Risk Survey [7].

Understanding patterns of socioeconomic difference in smoking over long periods with different stage of cigarette epidemic is important for public health. There are several studies examined socioeconomic gradients in smoking among different cohorts across time. Results from a trend study on smoking in Canada from 1950 to 2011 demonstrated that smoking rates have decreased over time but socioeconomic differences have increased which suggested that SES gradients emerge rapidly in later stages of the tobacco epidemic [24]. Furthermore, Vedoy found that the probability of daily smoking decreased faster across cohorts among higher compared to lower educated in accordance with the data on smoking in Norway from 1976 to 2010 [25]. However, it has also been reported in an American study that socioeconomic differences in smoking emerge across all birth cohorts investigated. Researchers found increasingly strong socioeconomic gradients in smoking across time especially among younger cohorts, which an inverse relation of SES to smoking was observed [26]. Thus, SES gradients in smoking with different stage of cigarette epidemic need to be addressed more explicitly.

Apart from different stages of cigarette epidemic, the socioeconomic differences in smoking of adolescents in developing countries are less studied comparing to studies done in developed country. And there are few studies exploring the associations between socioeconomic status and smoking between developing and developed countries with different stage of tobacco epidemic. The aims of this study, therefore, were to examine whether socioeconomic differences in adolescents’ smoking exist in China, representing the developing country in the third stage of cigarette epidemic, and Finland, representing the developed country in the final stage of cigarette epidemic, and then compare the relationships between SES and adolescents’ smoking in these two countries.

## Methods

This analysis used the HBSC data collected from Beijing, China and Finland. The same HBSC research protocol [27] was used in both countries. The target population of the HBSC is young people attending school aged 11, 13 and 15. The Chinese data were from the HBSC linked project survey in Beijing, which was conducted in December 2008. The Chinese survey was carried out by the China Institute of Sport Science (CISS) and it was approved by the ethics committee of the CISS. The Finnish data were obtained from the HBSC study 2006, conducted between March and May 2006. The Finnish HBSC study was carried out by the Research Centre for Health Promotion at the Department of Health Sciences of the University of Jyväskylä and it was approved by the Finnish National Board of Education and the Trade Union of Education in Finland.

### Sample

In China, the survey sampled from state schools all over the Beijing metropolitan area. Multi-stage stratified and random cluster sampling method was used to ensure the samples were weighted to be representative for Beijing metropolitan. Three stages were included during the sampling process: 1) Selection of the sample districts/counties; 2) Selection of the sample schools; and 3) Selection of the sample classes. In the first two each stages, stratified random sample method was employed to select the representative sample districts/counties and then the sample schools within them in terms of geographical location (urban or rural), economic development level, and school conditions [28]. At least three classes were chosen randomly from the sample schools. All students in the sample class were invited to the school survey. The response rates for schools and students in China were 91.5 % and 98.1 % respectively. The sample can represent the adolescents in Beijing, China.

The data of the Finnish part were obtained from the HBSC survey in Finland in 2006. During the sampling process, in order to select the representative sample at country level, the strata were decided based on all four provinces of Finland. Furthermore, the second level stratum divided those areas by urban and rural communities with the exception of the capital city area due to there being no rural communities. Hence, the final number of the strata used in the sampling procedure was seven. A special computer programme was used to choose the sample schools from the Finnish school register using cluster sampling with Probability Proportional to Size (PPS) of schools method (the size of schools was taken into account). One class was randomly selected within each sample school, except for only a few cases where more than one class were chosen from the same school in order to have enough representative participants in the sample. The response rate of sampled schools and students in Finland were 86.9 % and 88.2 % respectively.

Finally, a total of 6099 Chinese and 6046 Finnish students aged 11, 13, and 15 years took part in the abovementioned surveys. Due to the fact that smoking among 11- and 13-year-old students is rare in both countries, only 15-year-old participants were involved in the analyses (China: *N* = 2049; Finland: *N* = 2024). Samples were cleaned in line with the cleaning rule of the HBSC survey protocol if their gender and/or birth date were missing, or their age was beyond the target range [27]. The final data were used for analyses in present study including 2005 Chinese and 1685 Finnish 15-year-old pupils after cleaned for those not meet the research protocol (China: *n* = 30; Finland: *n* = 25) and non-response/missing values of the related items (China: *n* = 14; Finland: *n* = 314). Overall, the sample size and gender proportion were similar (Table 1) and there was no difference between the mean age of participants between two countries (China: 15.78 ± 0.32 years (mean ± SD); Finland: 15.78 ± 0.32 years (mean ± SD)). The detailed information of sampling procedure can be found elsewhere [29, 30].

**Table 1:** Socio-demographic characteristics of respondents in China (*n* = 2005) and Finland (*n* = 1685)

	China		Finland	
	n	%	n	%
Gender				
Boys	892	44.5	790	46.9
Girls	1113	55.5	895	53.1
FAS				
Low	631	31.6	519	31.6
Middle	666	33.3	687	41.7
High	701	35.1	439	26.7

### Instrument and variables

The HBSC survey was based on a self-completed questionnaire investigated during a normal school class. The students were instructed on how to fill in the questionnaire and also informed that only the researcher will read their answers. Students’ participation in the survey was voluntary and anonymous. The questionnaire used in the Chinese survey was based on the English version of the questionnaire used in the Finnish HBSC Survey in 2006. The questionnaire was firstly translated from English to Chinese by two researchers independently, and then translated from Chinese back to English by other professional translators to check for any discrepancies. The survey questionnaire has satisfactory test-retest reliability for the students in Beijing [31].

### Measurements of smoking

#### Ever smoked

Ever smoking was examined by asking the question ‘have you ever smoked (at least one cigarette, cigar or a pipeful)?’ with response options. The answers were: ‘yes’, or ‘no’. Respondents who answered ‘yes’ were defined as ever smoked.

#### Present smoking status

The present smoking status was assessed by asking the students ‘how often do you smoke at present?’ The response alternatives were: ‘every day’, ‘every week, but not daily’, ‘less than once a week’, ‘I do not smoke’. Weekly smoker was defined as those students reporting that they smoke every day or every week.

#### Onset of smoking

The initiation of smoking was examined by the item asking ‘at what age did you smoke a cigarette (more than a puff) for the first time?’ The answers were: ‘never’, ‘11 years old or less’, ‘12 years old’, ‘13 years old’, ‘14 years old’, ‘15 years old or older’. Early onset of smoking was defined as those respondents who reported first smoking at age 13 years or younger, the same cut-off used in the HBSC international survey report [32].

### Measurements of socioeconomic status

#### Family affluence scale

The ‘Family Affluence Scale’ (FAS) has been used to examine and explain socioeconomic inequalities in the HBSC study for more than 10 years, and it has also been proven as a reliable and valid SES measure for adolescents in China [29]. The items, response categories, codes and analyses strategy of FAS used in the present study are as follows:

“Does your family own a car, van or truck?”

Response categories were: No (=0); Yes, one (=1); Yes, two or more (=2).

“Do you have your own bedroom for yourself?”

Response categories were: No (=0); Yes (=1).

“During the past 12 months, how many times did you travel away on holiday with your family?”

Response categories were: Not at all (=0); Once (=1); Twice (=2); More than twice (=3).

“How many computers does your family own?”

Response categories were: None (=0); One (=1); Two (=2); More than two (=3).

A composite FAS score was calculated for each respondent based on his or her answers to these four items, ranged from 0 to 9. Three groups (low, middle and high) were categorized in terms of the composite FAS score. To get similar proportion of each category in both countries, different cut-offs were used for two countries respectively. For Chinese adolescents, FAS low (score = 0–2) indicated low affluence, FAS middle (score = 3–4) indicated middle affluence, and FAS high (score = 5–9) indicated high affluence, while for Finnish adolescents, low (score = 0–4), middle (score = 5–6), and high (score = 7–9) were calculated. The distribution of the FAS groups can be seen from Table 1.

### Statistical analyses

Descriptive statistics were used to show the characteristics of the study sample and the distribution of independent and dependent variables. Mean age of Chinese and Finnish participants were compared by Independent-Samples *T* test. Differences in percentage of smoking behaviour were compared by countries, and by gender and FAS for China and Finland respectively using Pearson’s Chi-square test. In addition, Pearson’s Chi-square test was also used to compare the prevalence of smoking behaviour of total population by country. Binary logistic regression models were used to investigate the relationships between all variables of smoking behaviour and FAS by gender for the two countries separately. The high FAS group was served as the reference category. Odds ratios with 95 % confidence intervals (CIs) were computed for other categories of independent variables. A *p* value of < 0.05 was considered statistically significant. All analyses were done by using Analytics Software (PASW, formerly SPSS), version 18.0 (SPSS, Inc., Chicago, Illinois, US).

## Results

In general, for all three variables of smoking behaviour, the results indicated that the prevalence was much higher among Finnish schoolchildren than Chinese schoolchildren (*p* < 0.001) (Table 2). Notably, the percentage of weekly smokers in Finland was nearly five times that in Beijing, China. Nearly one fifth (17.8 %) of Chinese adolescents reported they had ever smoked and 11.6 % smoked when they were 13 years or younger. For Finland, more than half (59.3 %) of students had smoked and 33.6 % were classified as early smokers. Among Chinese respondents, a gender difference was observed for all measures with boys exhibiting greater prevalence of smoking than girls (*p* < 0.001). In contrast, there was no difference in smoking behaviour between Finnish boys and girls.

**Table 2:** Prevalence of adolescents’ smoking by gender and by Family Affluence Scale (FAS) group in China (*N* = 2005) and Finland (*n* = 1685)

	Ever smoked	Weekly smoker	Early onset of smoking
	% (n)	% (n)	% (n)
China			
Total	17.8 (356)	4.6 (92)	11.6 (233)
*p* ^*a*^	<0.001	<0.001	<0.001
Gender			
Boys	29.7 (265)	9.7 (86)	19.6 (175)
Girls	8.2 (91)	0.5 (6)	5.2 (58)
*p* ^*b*^	<0.001	<0.001	<0.001
FAS			
Low	21.5 (135)	4.0 (25)	14.2 (88)
Middle	16.1 (107)	4.2 (28)	11.1 (72)
High	16.0 (112)	5.5 (38)	10.4 (72)
*p* ^*c*^	0.014	0.385	0.085
Finland			
Total	59.3 (1000)	21.9 (369)	33.6 (566)
Gender			
Boys	60.9 (481)	23.0 (181)	36.3 (287)
Girls	58.0 (519)	21.0 (188)	31.2 (279)
*p* ^*b*^	0.213	0.337	0.009
FAS			
Low	61.8 (320)	25.1 (130)	38.2 (192)
Middle	59.7 (410)	21.4 (147)	32.4 (218)
High	57.4 (252)	19.6 (86)	33.6 (145)
*p* ^*c*^	0.389	0.106	0.108

^a^Compared by two countries; ^b^Compared by gender group; ^c^Compared by FAS group

The statistically significant differences of the rates of having ever smoked among the different FAS groups were observed in China (*p* < 0.05). The highest percentage of those having ever smoked was found among Chinese respondents from the low FAS group (21.5 %) and the lowest percentage were from the high FAS group (16.0 %) (Table 2). Although the percentages of all three smoking indictors among Finnish students from the low FAS group were the highest, no statistically significant difference was found among different FAS groups (*p* > 0.05).

The logistic regression analyses of smoking and the FAS were performed in both countries stratified by gender (Table 3). When the smoking behaviour of students from the high FAS group were used as a reference group, we found that Chinese boys from the low FAS group were more likely to report having ever smoked (*OR* = 2.12, 95 % CI: 1.49–3.01), and being early onset of smoking (*OR* = 2.17, 95 % CI: 1.44–3.26) than those from the high affluence families. Finnish girls from the low FAS group were more likely to report being weekly smokers (*OR* = 1.68, 95 % *CI* = 1.07–2.65) compared to girls in the high FAS group. No significant difference was found for the relationships between FAS and smoking behaviour among Chinese girls and Finnish boys.

**Table 3:** The associations between adolescents’ smoking and Family Affluence scale (FAS) by gender in China (*n* = 2005) and Finland (*n* = 1685)

	China				Finland			
	FAS Low		FAS Middle		FAS Low		FAS Middle	
	OR	95 % CI	OR	95 % CI	OR	95 % CI	OR	95 % CI
Boys								
Ever smoked	2.12	1.49–3.01	1.22	0.85–1.74	1.06	0.72–1.55	0.98	0.69–1.39
Weekly smoker	0.83	0.48–1.43	0.82	0.48–1.39	1.17	0.77–1.79	0.82	0.54–1.24
Early onset of smoking	2.17	1.44–3.26	1.48	0.98–2.26	0.85	0.46–1.59	0.66	0.34–1.28
Girls								
Ever smoked	1.07	0.64–1.81	0.91	0.53–1.55	1.36	0.95–1.94	1.23	0.88–1.72
Weekly smoker	1.89	0.17–20.95	2.76	0.29–26.68	1.68	1.07–2.65	1.56	0.99–2.40
Early onset of smoking	1.19	0.81–1.75	0.82	0.57–1.19	1.28	0.88–1.87	1.09	0.76–1.58

The reference group in logistic regression was adolescents in the highest FAS group

## Discussion

With regard to the adolescents’ smoking, this study found that the prevalence of different patterns of smoking behaviour is much higher among Finnish adolescents than Chinese adolescents from Beijing. Among Chinese respondents, gender difference was observed for all four measures of smoking, with boys exhibiting greater prevalence of smoking than girls. In contrast, there were no differences of the percentage of ever smoked and being weekly smoker between Finnish boys and girls. The findings also revealed different socioeconomic differences in ever smoked and early onset of smoking among Chinese boys, and in weekly smoker among Finnish girls. No other statistically significant difference was found for the socioeconomic difference in smoking behaviour among Chinese girls and Finnish boys.

Although various results have been reported from previous studies concerning the socioeconomic inequalities in adolescents’ smoking, a majority of studies found that regular smoking was most prevalent among students from lower socioeconomic groups [15, 33]. However, it should be noted that most of those studies were mainly from western countries. For studies in developing countries, Doku and his colleague found that socioeconomic differences existed in smoking with higher prevalence in lower socioeconomic groups in Ghana [16]. In the present study, China represents the developing country and Finland represents the developed country. Our findings regarding Chinese adolescents from Beijing indicate that boys from a less affluent family are more likely to smoke and start smoke early. Meanwhile, with regard to Finnish results, it shows that female students from the low affluent families are at higher risk of being weekly smokers. It is in line with a study examined the socioeconomic differences in smoking among Finnish adolescents from 1977 to 2007, in which high rates of smoking were found in lower SES groups which persisted over time [34].

Smoking in adolescence is influenced by many factors. From the individual level, such as demographic factors (age, gender, ethnicity, SES), physiological differences (genetics, physiology reaction), and other risk behaviours, to the contextual level, such as peer relationships, parents and families’ smoking, environmental/culture context (tobacco advertising and media message, taxation, cost and policies) [35]. Thus, there are several plausible explanations for the different pattern of socioeconomic differences in smoking behaviour between Chinese and Finnish adolescents, as observed in our study.

Firstly, the possible explanation for the difference could be due to the different transition stages of smoking epidemic in Beijing, China and Finland. It was reported that the prevalence of smoking has been falling since the 1970’s in Nordic countries [36]. However, the smoking rates have not decreased simultaneously and equally in different socioeconomic groups. The rate of smoking among the higher social gradient has fallen more quickly, which leads to increasing inequalities of the smoking rate in different socioeconomic groups [37]. Previous studies on socioeconomic changes in adults’ smoking over long periods also reveal the fact that the socioeconomic differences increased though the prevalence of smoking decreased along with cigarette epidemic into the final stage [24].

Secondly, the changes of smoking prevalence might also happen differently between boys and girls in a certain stage, which contributed to the socioeconomic difference of smoking that observed among Finnish girls but not boys in current study because the changes (increase and/or decrease) of smoking prevalence among male population appear earlier than those changes among female population in terms of the four-stage model of smoking epidemic [2].

Thirdly, the absence of socioeconomic difference might be due to the fact that there has not been inequality in smoking during adolescence period or there has been a lack of valid measures of SES among adolescents to detect the difference. Adolescents’ SES is usually measured by using the information regarding their parents’ SES, such as parents’ education and occupation, and household income. However, one should be aware of the difficulties of measuring adolescents’ SES when using their parental SES as a proxy. Currie and her colleagues argued that it is still uncertain whether parents’ SES should be used as a proxy [20]. Furthermore, most previous studies regarding health inequality of adolescents were done in western countries and therefore the SES indicators should be proved to be valid in developing countries. For instance, Doku and his colleague tested Material Affluence Scale as a viable alternative method for measuring adolescent’s SES [16]. In present study, FAS, a proxy SES indicator used, has been proved a reliable and valid measure in SES of adolescent population in Beijing China [29], as well as in western countries [38–40].

The different gender-smoking relationships may also contribute the socioeconomic differences in smoking of Chinese and Finnish schoolchildren. The present study found that only very few Chinese girls have ever smoked or been being weekly smoker. Thus, the difference of smoking among Chinese girls from different SES groups may hardly, or even impossible, be found. However, the similar percentage of smoking behaviour among Finnish boys and girls provided the possibilities of indicating the difference in smoking from the different SES groups for both gender.

### Limitation

There are some methodological limitations regarding the present study should be addressed. Firstly, it should be argued that the current study used FAS as the only SES measure due to the lack of common SES indicators used in both Chinese and Finnish surveys. Although a variety of cross-national studies have been done to explicitly validate different aspects of the FAS [38–41], it has been suggested that perceived family wealth should be added as another item to FAS when comparison were made between countries [42]. Furthermore, the validity of FAS in a high-income and welfare state such as Finland has been contended since the FAS items were developed in the early 1990s and therefore they may not have enough power to distinguish different social gradients. In addition, despite the fact that the FAS items examine the material wealth including bedroom, computer, car and holidays, the culture meaning of these items presenting behind may differ in China and Finland.

Secondly, following the HBSC research protocol [27], the current study used cluster sampling method and the basic unit is class, which may cause higher standard errors compared to a sampling of individuals. The data structure of the sample unit being class rather than student may generate larger confidence interval and therefore a misleading interpretation of the results, especially if the *p* value is near 0.05.

Finally, it should be pointed out that another methodological limitation concerning the comparability in the present study is that the Chinese data were only sampled from the Beijing area due to the aims of the survey and the limited resources and financial support. In a country with huge diversity such as China, the results should be interpreted with caution since the sample cannot represent the whole nation although the prevalence of smoking in the current study is very similar to the national estimates [7]. It is true that conclusions cannot be drawn based on the comparison of the prevalence of youth substance use. However, it is possible that the patterns of socioeconomic inequalities in adolescents’ smoking in Beijing, as a reflection of such patterns in a Chinese population, can be compared with the patterns of other countries or regions. Nevertheless, the results of the comparison should be discussed and interpreted with caution. In addition, the present study only compared one high-income and one developing country. Comparison between more countries should be encouraged in future studies to ensure the relationship is largely due to SES and not other environmental or cultural factors.

## Conclusions

In conclusion, different patterns of socioeconomic difference in smoking between Chinese and Finnish adolescents were observed by gender and by smoking behaviour in the present study. Chinese boys from the low affluent families were more likely to being smokers and starting smoke early, while Finnish girls from less affluent families were more likely to be weekly smokers. The present study suggests that socioeconomic differences in smoking are different among adolescents in countries with different stage of smoking. Country specific policies and interventions for different target groups should be encouraged and designed for reducing the prevalence of adolescents’ smoking.

## Abbreviations

AHLS: Adolescent Health and Lifestyle Survey
CI: Confidence Interval
CISS: China Institute of Sport Science
ESPAD: European School Survey Project on Alcohol and Other Drugs
FAS: Family Affluence Scale
HBSC: Health Behaviour in School-aged Children
OR: Odds Ratio
PPS: Probability Proportional To Size
SES: Socioeconomic Status
